# Experience real-time, health and biological outcoMes of personal recovery in PeOple With mEntal disorders in Residential facilities (EMPOWER): a cohort study

**DOI:** 10.1192/j.eurpsy.2024.1486

**Published:** 2024-08-27

**Authors:** A. Martinelli, G. de Girolamo

**Affiliations:** IRCCS Istituto Centro San Giovanni di Dio Fatebenefratelli, Brescia, Italy

## Abstract

**Introduction:**

Deinstitutionalization has resulted in diverse mental health care models, influenced by local resources, funding, and cultural factors. In Italy, 127 Department of Mental Health (DMHs) provide care for individuals with mental disorders. People with severe mental disorders (SMD) live independently or in residential facilities (RFs). Approximately half of the Italian DMH budget is allocated to RFs, serving around 3.6% of people with SMD. Italian RFs prioritize personal recovery, empowering individuals with SMD to live fulfilling lives despite symptoms and psychosocial challenges. While personal recovery is known to improve well-being and cost-effectiveness, its implementation in Italian RFs remains incomplete. There is insufficient evidence regarding its impact on various outcomes for residents, including health, psychosocial, and biological factors.

**Objectives:**

The EMPOWER Study aims to assess whether adding personal recovery to Treatment As Usual (TAU) in Italian RFs could improve functioning (primary outcome), health, biological status, productivity and interpersonal relationships (secondary outcomes) among patients receiving the personal recovery-oriented treatment, compared with TAU. Additionally, data will be collected from informal caregivers, mental health professionals, and concerning the recovery orientation of RFs.

**Methods:**

This study employs a longitudinal cohort design, gathering data at baseline and six-month follow-up in Italian RFs. A cohort of residents over 18 y.o. who receive a personal recovery-oriented treatment, the Mental Health Recovery Star (N=20), is compared to a matched group of residents receiving the TAU (N=20). International standardized assessments collect patients’ data on functioning, psychopathology, need for care, quality of life (QoL), positivity, social network, service satisfaction, and patient stigma. Informal caregivers’ data includes burden, QoL, positivity, and service satisfaction. Mental health professionals’ data encompasses burnout, stress, stigma, positivity, and work satisfaction. The working alliance between professionals and patients is assessed. Clinical and biological exams (blood and saliva samples) are collected, along with actigraphy data on patients’ circadian rhythm and physical activities. Digital data through a mobile app captures psychopathology, productive activities, social network, using the Experience Sampling Method with questions defined with patients. Focus groups with patients, professionals, and informal caregivers are facilitated by an expert by experience. Recovery orientation of RFs is assessed.

**Results:**

Not yet available.

**Conclusions:**

This study aims to generate novel insight that could improve our treatment approaches for patients in residential facilities.

**Disclosure of Interest:**

None Declared

